# Targeted Complement Treatments in Glomerulopathies: A Comprehensive Review

**DOI:** 10.3390/jcm14030702

**Published:** 2025-01-22

**Authors:** Micaela Gentile, Lucio Manenti

**Affiliations:** 1 UO Nefrologia, Dipartimento di Medicina e Chirurgia, Università di Parma, 43126 Parma, Italy; migentile@ao.pr.it; 2 Nephrology Unit, Azienda Sociosanitaria Liguria 5, 19121 La Spezia, Italy

**Keywords:** complement system, thrombotic microangiopathy, glomerulopathy

## Abstract

The complement system includes soluble and cell surface proteins and is an important arm of the innate immune system. Once activated, the complement system rapidly generates proteins with inflammatory and vasoactive activities. Although complement is crucial to host defense and homeostasis, its inappropriate or uncontrolled activation can also drive tissue injury. Glomerulopathy encompasses a spectrum of diseases with diverse etiologies, clinical presentations, and outcomes. Among the intricate web of factors contributing to glomerulopathies pathogenesis, the role of complement activation has emerged as a focal point of research interest and therapeutic intervention. The pioneer drug was eculizumab, which made it possible to drastically change the prognosis of atypical hemolytic uremic syndrome, an otherwise fatal disease. This comprehensive review aims to elucidate the multifaceted interplay between complement pathways and glomerulopathies, shedding light on potential pathways for targeted therapies and improved patient care.

## 1. Introduction

The complement system, an integral component of innate immunity, comprises a cascade of proteins that orchestrate immune responses against pathogens while maintaining self-tolerance. However, dysregulation of complement activation can precipitate tissue damage and inflammation, contributing to the pathogenesis of various kidney disorders [1]. The contributions of complement dysfunction range from primary cause to secondary driver of disease progression. The unique susceptibility of the kidney to complement-mediated injury may be due to several factors, including a high concentration of complement proteins in the glomerular capillaries due to the filtration of plasma in glomerular capillaries, exposing the glomerular basement membrane (GBM) to a high concentration of complement proteins. In addition, the GBM does not express intrinsic complement regulators [2,3].

In the last decade, emerging studies have revealed the role of the complement system in a wide range of kidney disorders [4], such as atypical hemolytic uremic syndrome (aHUS), membranoproliferative glomerulonephritis (MPGN, including C3 glomerulopathy and lupus nephritis), IgA nephropathy (IgAN), membranous nephropathy (MN), and unexpected diseases such as ANCA (anti-neutrophil cytoplasmic antibodies)-associated vasculitis (AAV), showing the role of complement in disease pathophysiology.

Recent advancements in our understanding of complement biology have unveiled intricate regulatory mechanisms and complex crosstalk with other immune and inflammatory pathways, both systemic and renal [5]. Notably, the complement cascade crosses with pathways involving immune complexes, autoantibodies, and cytokines, amplifying kidney injury and perpetuating chronic damage until end-stage kidney disease (ESKD).

The complement system also has inflammatory and metabolic functions that seem to be involved in the pathogenesis of atherosclerosis of vessels and promoting local inflammation with the amplification of the inflammatory response and the recruitment of immune cells [6]. Several growth factors and cytokines, such as tumor growth factor-**β1** (TGF-β1), fibroblast growth factors (FGFs), matrix metalloproteinases (MMPs), and tissue inhibitors of metalloproteinases (TIMPs), act as pro-inflammatory agents, influencing complement activation [7,8].

Moreover, genetic factors and environmental triggers may increase the risk of complement dysregulation, worsening kidney damage and influencing disease progression. Even individuals with multiple genetic predispositions often need an external stimulus, such as infections, pregnancy, organ ischemia, or medications, for the disease to manifest [9]. Advances in genetic and molecular research have highlighted key complement components and regulators as potential biomarkers for disease activity, progression, and therapeutic outcomes in nephropathies. This review aims to explore the complex interactions between complement pathways and glomerulopathies, exploring in depth the role of complement in the pathogenesis of each kidney disease, offering insights into potential therapeutic targets and future improvements in patient care (Table 1).

## 2. The Complement Cascade

**Figure 1:**
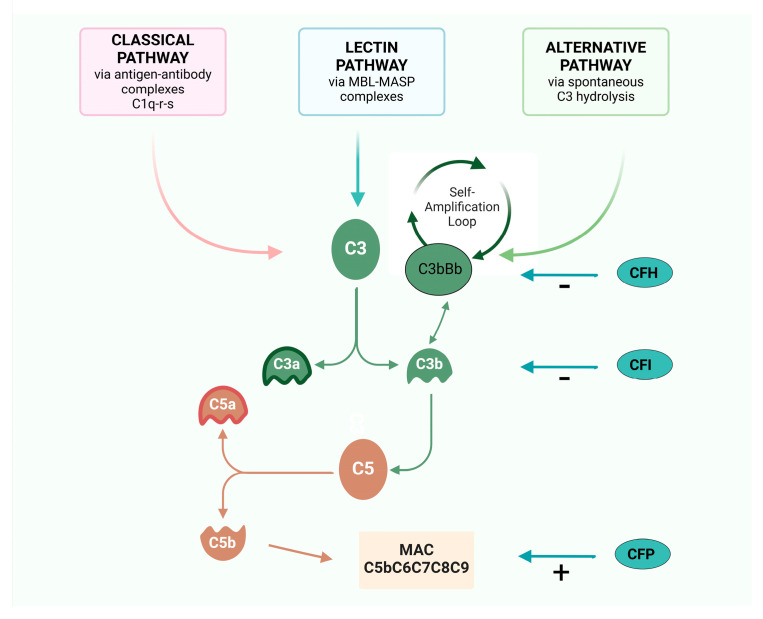
The three pathways of the complement system: classical, lectine, and alternative. The figure emphasizes the importance of C3 and the factors that act in reducing or enhancing the activation of the cascade until the formation of the MAC.

Comprising a cascade of soluble and membrane-bound proteins, the complement system operates through three distinct activation pathways—classical, lectin, and alternative—converging at the formation of effector molecules that mediate immune functions, inflammation, and immune surveillance (Figure 1). The complement system interfaces with several immune and non-immune pathways, exerting pleiotropic effects on coagulation pathway, host defense, tissue development, and homeostasis [10].

Each pathway takes place in response to unique molecular signals, but they all converge to catalyze the proteolytic cleavage of C3, through the C3 convertase, into two fragments: C3a and C3b. This cleavage is essential to the formation of the membrane attack complex (MAC) resulting in opsonization, inflammation, and cells lysis [11].

The classical pathway (CP) is triggered by the binding between antigen and antibody to form an immune complex: the Fc fragment of IgG or IgM binds C1q with proteinases C1r and C1s, forming the complex C1q-r-s.

The lectin pathway (LP), instead, is activated by mannose-binding lectins (MBL) that recognized pathogen-associated molecular patterns (PAMP) and altered self-antigens.

The activation of these two pathways converts on the formation of classical C3 convertase (C4b2a convertase) [12], which consists of two fragments: C4b and C2a. This complex cleaves C3 into C3a and C3b upon binding to C3. C3b plays a role in the formation of alternative C3 convertase (C3b-Bb convertase) and C5 convertase (C3 convertases-C3b).

The alternative pathway (AP), instead, is spontaneously activated: the alternative C3 convertase (C3b-Bb convertase) is generated spontaneously through low-level hydrolysis of C3 and consists of two fragments: C3b and factor Bb. This complex is stabilized by properdin (factor P) and constantly triggered to cleave additional C3 molecules, amplifying the complement cascade, but controlled by several factors—the most important factor B, factor I, and factor H—that avoid its uncontrolled activation [13].

Apart its role in the initial activation steps, C3b has three additional important functions:

- it binds to cell surfaces in complex with factor B, triggering a positive feedback loop (called an “amplification loop”) with the generation of even more C3a and C3b, leading to rapid amplification of the AP [14];
- it promotes the formation of the C5Conv, leading to the formation of the MAC and C5a;
- it operates as an opsonin, coating foreign particles and facilitating the recognition and phagocytosis of pathogens by immune cells such as macrophages and neutrophils [14].

Thus, the C3 convertases system serves as a central hub and C3b is the leading complement fragment that facilitates the complement cascade, initiating the amplification loop of complement activation [13].

C3a and C5a act as potent anaphylatoxins through the recruitment of leukocytes at the site of inflammation, also promoting the synthesis and release of granules consisting of enzymes and oxidizing agents [14].

In summary, complement antagonizes pathogens through the formation of mediators that increase the ability of phagocytosis through C3b-mediated opsonization, promoting inflammation through C3a and C5a anaphylatoxins, and causing lysis of pathogens through the formation of the MAC complex [14].

## 3. Regulator Proteins

Such a violent inflammatory activation system requires very fine-tuned regulation, for which there are numerous, incompletely understood, regulatory proteins, many of which modulate the central C3 convertase.

Factor H (CFH) is the principal negative regulator of the AP [14]. This plasma protein controls complement activation in multiple ways, such as promoting the decay of the C3bBb convertase, encouraging the detachment of C3b from the host cell surface, and acting as a cofactor for Factor I (CFI) [15]. CFH is able to discriminate between healthy host cells and altered host cells or pathogens through binding its C-terminal domains to specific glycan [16,17]. Moreover, by blocking further activation and amplification of the AP, CFH also downregulates the LP and CP. Factor H-related proteins (FHRs) exhibit structural similarity to CFH, comprising multiple complement control protein (CCP) domains and a C-terminal domain. Like CFH, FHR proteins contain binding sites for complement components (such as C3b) and glycosaminoglycans (GAGs), enabling them to interact with complement proteins and regulate complement activation. It has been shown that some FHRs, such as FHR-1, FHR-2, and FHR-5, compete with CFH for binding to C3b and to other complement components. This competitive binding may influence the balance of complement activation and inhibition [18]. CFI acts as a key regulator of the alternative pathway by cleaving and inactivating C3b into its inactive form (iC3b), preventing the assembly and stabilization of the alternative pathway C3 convertase, thereby inhibiting further amplification of the complement cascade [19].

Properdin (CFP) stabilizes the alternative pathway C3bBb convertase, but not the classical/lectin pathway C4b2a convertase [20]. In contrast to its pro-inflammatory role as a stabilizer of C3bBb convertase, CFP also shows regulatory functions by facilitating the decay of C3 convertases and promoting the clearance of complement-opsonized immune complexes [20].

Complement Receptor 1 (CR1) is expressed on erythrocytes and immune cells; CR1 regulates complement activation, acting as a cofactor for CFI-mediated cleavage of C3b and accelerating the decay of C3 convertase. CR1 also facilitates the clearance of complement-opsonized particles and immune complexes, thereby controlling the intensity and duration of complement-driven immune responses. Membrane cofactor protein (MCP, CD46) interferes with C3 convertase assembly by facilitating CFI-mediated C3b inactivation [21]. Decay-accelerating factor (DAF, CD55) avoids complement activation on self-cells by promoting C3 and C5 convertase decay [22]. Protectin (CD59) binds C9, consequently preventing MAC formation [12]. Beyond soluble inhibitors and membrane-bound regulators, regulatory T cells (Tregs) exert immunomodulatory effects on complement activation and inflammation through cell–cell interaction and cytokine secretion. Tregs suppress excessive complement activation and mitigate tissue injury by promoting immune tolerance and dampening inflammatory response, thereby maintaining immune homeostasis [23]. Dysregulation of complement control can lead to autoimmune diseases, inflammatory conditions, and tissue damage, highlighting the need for strict regulation of complement dynamics. Recent research into the complement system’s role in various diseases has driven the development of novel therapeutic agents targeting different complement components (Table 1).

## 4. The Role of Complement in Kidney Disease

### 4.1. Atypical Hemolytic Uremic Syndrome

Thrombotic microangiopathy (TMA) characterizes diseases with severe vascular endothelium damage mediated by different pathogenetic pathways, following pro-inflammatory and pro-coagulant changes in the endothelium leading to intravascular thrombi, ischemia, and organ dysfunction [24,25]. Microangiopathic hemolytic anemia, thrombocitopenia, elevated lactate dehydrogenase (LDH), undetectable serum haptoglobin, and the presence of schistocytes in peripheral blood smears are easily detectable in blood tests [26]. Not all patients with TMA have hematologic abnormalities; sometimes TMA can be detected on histologic lesions, in particular in kidney biopsies where the most characteristic acute TMA’s lesions are thrombi occluding vessels with endothelial swelling [27]. TMA can lead to death or organ dysfunction. It is essential to perform a differential diagnosis to identify the type of TMA.

Hemolytic uremic syndrome (HUS) represents the systemic expression of TMA. In particular, primary atypical hemolytic syndrome (aHUS) is an exclusion diagnosis after ruling out Shiga toxin-producing *E. coli*-associated HUS, thrombotic thrombocytopenic purpura, and secondary etiologies [28]. About 60% of aHUS are mediated by pathogenic variants or antibodies against complement components [29], in particular C3, CD46, CFB, CFH, CFHR5, CFI, THBD, and anti-FH antibodies [30].

In general, it is estimated that 50 percent of cases progress to ESKD, with mortality rates around 25 percent; these figures also vary depending on extra-renal involvement and the latency between the onset of symptoms and signs and the beginning of therapy. Treatment with eculizumab, compared to plasmapheresis, has radically changed the prognosis of these patients: prospective studies [31,32], confirmed by retrospective and clinical cases [33], have showed a rapid improvement in hematological abnormalities and progressive recovery of kidney function in most patients.

Eculizumab is the first marketed drug targeting a specific complement molecule. It is a humanized IgG2-IgG4 hybrid monoclonal antibody (Soliris, Alexion Pharmaceuticals) which binds the C5 complement protein with high affinity and blocks the generation of the pro-inflammatory molecules C5a and C5-b9 [31]. Eculizumab was first approved for paroxysmal nocturnal hemoglobinuria (PNH) and only later for use in atypical hemolytic uremic syndrome (aHUS). The rationale for its introduction in aHUS lies in the fact that the disease is secondary to an altered regulation of the complement pathway and is proven to occur in at least 50% of cases. The two prospective phase 2 studies (NCT01194973, NCT00844545) that led to its approval demonstrated the efficacy and safety of eculizumab in both plasmapheresis non-responsive and plasmapheresis-dependent patients. Moreover, there was a significant recovery of kidney function, with suspension of dialysis in 80% of cases. Another study also showed that eculizumab was effective in preventing post kidney transplantation aHUS recurrence [34], thus making it possible to transplant aHUS patients, who otherwise were considered to be at too high risk of disease recurrence. These results determined the FDA and EMA approval of its use despite the study being a phase II, non-randomized trial. In clinical practice, eculizumab demonstrated the ability to radically change the prognosis of aHUS patients.

Eculizumab is administered via intravenous infusion. The initial dose is 900 mg every week followed by maintenance doses of 1200 mg given every 2 weeks. The interruption of the therapy must be carefully evaluated, considering genetic predisposition, the severity of the disease, and the clinical course.

Blocking the terminal part of complement is also thought to be effective in promoting recovery of function in all secondary forms of TMA. Unfortunately, no studies have been designed to validate this valuation from the use of the drug in a real-life setting. In particular, evidence has accumulated on TMA secondary to lupus nephritis, renal scleroderma crisis, ANCA vasculitis, IgA nephropathy, malignant hypertension, and antiphospholipid syndrome [24,35].

Secondary forms of TMA may initially present as kidney-limited forms, making their classification as TMA more difficult and delaying the initiation of specific therapy [36]. Consensus conferences should be undertaken to establish guidelines to provide a shared approach for these particular conditions. It should also be emphasized that, in the course of TMA secondary to lupus nephritis (LN) and anti-phospholipid antibody syndrome, there is sufficient agreement for the use of eculizumab to rapidly extinguish the TMA and pending treatment of the cause of the secondary TMA, particularly in plasmapheresis-resistant TMA [37].

In recent years, ravulizumab has been developed, the first and only long-acting C5 complement inhibitor able to providing an 8-week duration of action, compared to every two weeks with eculizumab. Clinical trials documented ravulizumab’s non-inferiority to eculizumab in treating PNH and aHUS. This drug, which has a longer half-life, allows for an improvement in quality of life by limiting the frequency of e.v infusions.

The phase 3 studies evaluated the efficacy and safety of ravulizumab (NCT03131219, NCT02949128) in complement inhibitor-naïve adults and adolescent who fulfilled the diagnostic criteria for atypical hemolytic uremic syndrome. Ravulizumab treatment resulted in an immediate, complete, and sustained C5 inhibition in all patients. Normalization of platelet count, serum lactate dehydrogenase, and hemoglobin observed in the 26-week initial evaluation period [38] was sustained until the last available follow-up, as were the improvements in the estimated glomerular filtration rate (eGFR) and patient quality of life [39].

The anti-C5 monoclonal antibodies therapy altered the immune response encapsulated bacteria, particularly to Neisseria infections [40]. Thus, disseminated gonococcal infection has been reported in patients treated with eculizumab, and the risk of meningococcal infection has increased up to 10,000-fold following eculizumab treatment. Consequently, meningococcal vaccination and antibiotic prophylaxis are recommended for patients receiving eculizumab/ravulizumab [40]. However, susceptibility to meningococcal infection rate decreased over time [40]; the related mortality remained steady. Continued awareness and implementation of risk mitigation protocols are essential to minimize the risk of meningococcal and other Neisseria infections in patients receiving anti-C5 monoclonal antibodies.

Studies to evaluate the efficacy of iptacopan (an oral complement Factor B inhibitor, NCT04889430, NCT05795140, NCT05935215), crovalimab and nomacopan (a new C5 inhibitors, NCT04861259, NCT04958265, NCT04784455) are ongoing. A phase 2 study is ongoing to evaluate Pegcetacoplan (C3 inhibitor, NCT05148299) in transplant-associated-TMA after hematopoietic stem-cell transplantation.

### 4.2. Membranoproliferative Glomerulonephritis

Membranoproliferative glomerulonephritis (MPGN) stands as a formidable entity among the spectrum of glomerular diseases, characterized by distinctive histopathological features and different clinical manifestations. Histologically, MPGN manifests as mesangial proliferation, capillary wall thickening and double contour formation, reflecting the underlying immune-mediated injury to the glomerular basement membrane [41]. Clinical presentation of MPGN spans a spectrum ranging from asymptomatic proteinuria and hematuria to progressive kidney insufficiency and nephrotic syndrome, which poses diagnostic challenges, requiring a comprehensive evaluation to elucidate the underlying etiologies and guide therapeutic interventions. While kidney biopsy remains the cornerstone of diagnosis, serological and genetic studies play a complementary role in outlining the underlying pathogenic mechanisms and prognosticating disease outcomes [42].

MPGN has recently been reclassified on an etiopathogenetic basis, revolutionizing the previous approach that described histopathological aspects that often overlapped and did not allow for an appropriate subdivision with respect to the cause [43]. This new classification distinguishes MPGN into two main groups: complement-mediated (named C3 glomerulopathy) and immune complex-mediated (IC-MPGN).

According to this classification, C3 glomerulopathy (C3G) is characterized by immunofluorescence recognition of isolated glomerular C3 deposits and includes C3 glomerulonephritis and dense deposit disease. Acquired factors are involved in most patients, namely autoantibodies that target the C3 or C5 convertases. These autoantibodies drive complement dysregulation by increasing the half-life of these vital but normally short-lived enzymes. Genetic variations in complement-related genes were identified in about 25% of patients, in particular of C3, CFB, but also CFH, CFI, DGKE, and CFHR genes.

When immunofluorescence shows complement and immunoglobulin deposition, the disease is considered to be IC-MPGN. Rarely, a histological MPGN pattern is documented in the absence of deposits on immunofluorescence staining, which should lead to suspicion of a chronic TMA [44].

Therapeutically, the management of MPGN remains challenging, with no consensus guidelines and limited evidence-based interventions to guide clinical practice. Immunosuppressive regimens, including corticosteroids, cytotoxic agents, and rituximab, have been employed empirically in selected cases, aiming to modulate immune-mediated inflammation and halt disease progression. However, the variable response to immunosuppressive therapies underscores the need for tailored approaches informed by disease phenotype, complement profile, and genetic predisposition [45].

Regarding C3G, the efficacy of eculizumab was evaluated in a small clinical trial involving six patients: two patients showed an improvement in proteinuria, one showed complete remission, and one patient showed stabilized kidney function [46]. On the basis of this results, eculizumab in C3G was not implemented any more.

Recently, new complement-targeted treatments were attempted in C3G. The phase 3 APPEAR-G3G study (NCT04817618) evaluated the efficacy of iptacopan in patients with C3G. This study has showed a normalization of plasma C3, a reduction in proteinuria, and an improvement in the estimated glomerular filtration rate (eGFR) [47].

The randomized study ACCOLADE (NCT03301467) evaluated the use of avacopan (C5 receptor inhibitor), showing a reduction in histologic disease chronicity compared to placebo but failing to achieve a reduction in disease activity [48,49]. The two phase 3 studies VALIANT (NCT05067127) and VALE (NCT05809531) are evaluating the use of pegcetacoplan. Preliminary results have showed a reduction in proteinuria and stabilization of kidney function. The phase Ib study NCT05647811 will evaluate the safety, efficacy, and immunogenicity of the molecule NM8074 (Factor Bb inhibitor) administered intravenously to patients with C3 glomerulopathy, while the phase 2 study NCT06209736 will evaluate zaltenibart (MASP3 inhibitor).

### 4.3. Secondary IC-MPGN (Lupus Nephritis)

Regarding the immune complex-mediated glomerulonephritis, complement plays a pivotal role in lupus nephritis (LN). Indeed, complement has a biphasic nature, known as the “lupus paradox”: complement activation due to the deposition of immune complexes causes tissue damage, whereas genetic deficiencies of the early components of complement activation pathway (C1q and C4) can lead to the development of the disease. All the three pathways are involved in LES, so complement serum levels (C3, C4, CH50, C1q) and complement deposition on histological tissues are used for the management of SLE [50]. Rossi et al. showed that low C3 serum is associated with a high risk of ESKD [51].

It must be emphasized that, in approximately 5–15% of cases, TMA can be present in kidney biopsies of patients with LN, with the worst outcome. To date, there have been no clinical trials on the use of eculizumab in LN; however, it is now established practice to introduce eculizumab considering complement-mediated microangiopathic damage prevalent over proliferative inflammatory damage secondary to immune-complex deposition [52].

Randomized trials are ongoing to evaluate the efficacy of pegcetacoplan, iptacopan (NCT05268289), and ravulizumab (NCT04564339) [53]. Moreover, a phase 1b trial to assess the safety, tolerability, and pharmacodynamics of ANX009 (NCT05780515), a C1q inhibitor, is underway.

### 4.4. IgA Nephropathy

IgA nephropathy (IgAN) is the most common primary glomerulonephritis worldwide. It was first described by Jean Berger in 1969 [53] and has an annual incidence of 2–10 cases per 100,000 people. The classical phenotype includes hematuria intercurrent with gastrointestinal or upper respiratory tract infection. In particular, gross hematuria is more typical in children while asymptomatic hematuria with various degrees of proteinuria is the most common presentation in adults [54]. The progression of the disease is usually slow, but up to 20–40% of patients develop end-stage kidney disease (ESKD) within 20 years after diagnosis [55].

The pathogenesis is based on the “four-hit-hypothesis” characterized by the increased circulating levels of an aberrantly glycosylated galactose-deficient IgA (gd-IgA1), followed by the formation of immune complexes with an anti-gd-IgA1 antibodies with the deposition in the glomerular mesangium, leading to kidney injury. However, the presence of gd-IgA1 is by itself not sufficient to cause IgAN [56]. Several studies have showed the involvement of AP and LP in IgAN [57] and the presence of complement components in kidney biopsies may distinguish between IgAN and IgA deposition that can be found in healthy subjects. C3, CFH, properdin, and CFH-related proteins are found in kidney biopsies [57]. In particular, glomerular C3 deposition [58] and the reduction in the serum level of C3 [59] seem to correlate with the progression of the disease [60].

The finding of C4d in the absence of C1q in kidney biopsies suggests that the LP is also involved in the pathogenesis of the disease. Gd-IgA1 may trigger LP activation through the interaction with ficolin [57]: increased circulating levels of ficolin, MBL-associated protease (MASP-1), and MBL-associated protein (Map-19) were found in IgAN compared to healthy controls [61].

The evidence of the involvement of complement in IgAN has resulted in the beginning of several clinical trials to evaluate the efficacy of direct molecules against complement components [62].

Recently, iptacopan (NCT04557462) showed superiority in reducing proteinuria by 38% at 9 months follow-up over placebo in an interim analysis of a study involving more than 200 patients [63]. Data about the permanence of the effect on proteinuria reduction and kidney function are expected with the conclusion of the study. Lafayette et al. [63] showed an improvement in proteinuria and stability of eGFR in high-risk patients with advanced IgAN treated with narsoplimab (OMS721, MASP2 inhibitor, NCT02682407) [64]. However, the interim analysis of the ARTEMISAN-IGAN (NCT02682407) study failed to show any results on proteinuria reduction and the study has recently been stopped [65] (Omeros (2023). Press release: Omeros Corporation provides update on interim analysis of ARTEMIS-IGAN Phase 3 trial of narsoplimab in IgA nephropathy).

Recently, a small phase 2 study testing cemdisiran (NCT03841448), a new anti-C5, in nine patients over placebo in IgA nephropathy showed more than a 30% reduction in proteinuria 32 weeks after treatment [66]. Phase 2 studies for the use of pegcetacoplan (NCT03453619) and IONIS-FB-LRX (Factor B inhibitor, NCT04014335) in patients with IgAN are ongoing, while NM8074-IgAN-601 (NCT06454110) will study the effect of NM8074 on reducing proteinuria in IgAN patients.

The activation of AP and LP converge to the terminal pathway. C5aR knockout mice had less proteinuria, C3, and IgA deposition in the glomeruli. In humans, MAC glomerular deposition has a positive correlation with the degree of glomerulosclerosis, tubular atrophy, and interstitial inflammation in IgAN [62]. However, the use of eculizumab has not shown efficacy in patients with IgAN [67]. Nevertheless, a phase 3 study for the use of ravulizumab is ongoing (NCT06291376). Differently, a pilot phase II study (NCT02384317) showed the positive effect of avacopan, with an improvement in proteinuria after 12 weeks of treatment [68].

### 4.5. ANCA-Associated Vasculitis

ANCA-associated vasculitis (AAV) represents a group of systemic autoimmune diseases characterized by necrotizing inflammation of small to medium-size blood vessels without immune-complex deposition, and by the presence in the serum of anti-neutrophil cytoplasmic antibodies (ANCA), especially anti-proteinase 3 (PR3) and anti-myeloperoxidase (MPO). AAV includes microscopic polyangiitis (MPA), granulomatosis with polyangiitis (GPA), eosinophilic granulomatosis with polyangiitis (EGPA) and kidney-limited vasculitis [69]. Vasculitis is characterized by a heterogeneous clinical presentation with not specific constitutional symptoms (fatigue, fevers, weight loss). Virtually all organs can be involved. The involvement of the upper respiratory tract is more common in GPA than MPA; the lung involvement is characterized by alveolar hemorrhage in MPA, whereas pulmonary necrotizing granulomatous lesions are more common in GPA [70]. Kidney involvement is common and can lead to end-stage kidney disease if not early recognized. The introduction of immunosuppressive therapy has improved survival: 90% of patients achieve remission [71], but roughly 50% of patients relapse [72].

The absence or paucity of immunoglobulins and complement deposition in kidney biopsies have suggested, in the past years, the lack of involvement of the complement cascade in the pathogenesis of AAV [73]. Only in 2007, Xiao et al. [74] investigated the possible role of complement in AAV and showed the importance of complement activity, especially of the AP. Neutrophils activated by ANCAs release properdin that activates the alternative pathway with the generation of C5a. C5a binding to the specific receptor (CD88) on the neutrophils surface generates an amplification loop of the inflammatory process until the development of characteristic necrotizing lesions. C5a also contributes to the formation of MAC complex with cell lysis and further endothelial damage [75]. An association was observed between hypocomplementemia (serum C3) and more severe kidney involvement and worst outcome, suggesting an active role of AP in kidney damage [76].

The association between AAV and TMA was considered rare until a few years ago [77]. Chen et al found a prevalence of TMA of 13.6% in patients with vasculitis, with a higher risk of worse kidney outcome [77]. An abnormal activation of the AP can occur in AAV with positive inflammatory feedback loop between ANCAs, neutrophils, and complement [78]. It results in endothelia damage, especially due to C5a, and microangiopathic lesions. In addition, a strong association has been shown between low serum C3 and histologic signs of TMA with a worse kidney prognosis [79]. Although eculizumab has not been tested in AAV, but only used in sporadic clinical cases with good efficacy [80,81], the importance of the role of complement was showed in recent years by the efficacy of a new complement-targeted oral drug, a C5a receptor inhibitor, avacopan (CCX168). The therapeutic effects of avacopan are attributed to the inhibition of C5aR activity on neutrophils. The efficacy was showed not only in vitro [82] but also in vivo: in a phase II trial, improved kidney function has been demonstrated, decreasing the need for glucocorticoids and maintaining a sustained remission after 52-week respect steroid [83]. Moreover, the latest phase 3 clinical trial, ADVOCATE study (NCT02994927), compared AAV patients treated with standard of care (rituximab or cyclophosphamide) plus avacopan (suspension of steroids immediately) versus standard of care (with a 26 weeks tapering schedule of prednisone) in patients with AAV treated with concomitant immunosuppressive regimens.

The study enrolled more than 300 patients and showed non-inferiority of avacopan compared to steroid at weeks 26 and 52 (*p* value < 0.001) in obtaining vasculitis remission, whereas superiority at week 52 (*p* value = 0.007) was documented in maintaining sustained remission. Less adverse events were observed in the avacopan group compared the steroid group [83]. This relevant study led to the approval by the US and European drug regulatory agencies of avacopan in the treatment of ANCA-associated vasculitis. Furthermore, it was shown that patients treated with avacopan had a greater recovery of kidney function than those treated with the steroid. One of the limitations of the study was that patients with end-stage kidney disease (eGFR < 15 mL/min) were not enrolled, which may limit its use in cases of severe AAV and progressive kidney involvement. However, recently a post-hoc analysis of the ADVOCATE study showed that the benefit of avacopan in kidney functional recovery appears to be even greater in cases with more aggressive kidney involvement.

A phase 2 trial with Vilobelimab, a monoclonal antibody targeting C5a, was reported by the manufacturer as having terminated in 2021 with positive results (NCT03895801, NCT03712345). However, the study has not been published yet and there is no information about the start of an ongoing phase III study. A phase 2 study for the use of iptacopan (NCT06388941) is ongoing.

### 4.6. Membranous Nephropathy

Membranous nephropathy (MN) is the most common cause of nephrotic syndrome in adults characterized by thickening of the glomerular basement membrane due to immune-complex deposition resulting in structural and functional damage of podocytes [84].

One third of patients go into spontaneous remission, while 40% of patients develop ESKD within 10 years after diagnosis. Some 80% of MN are idiopathic, characterized by the presence of autoantibodies against podocyte antigens PLA2R (phospholipase A2 receptor 1) and THSD7A (thrombospondin type-1 domain-containing protein 7A) [85]. Some patients, without anti-PLA2R/THSD7A antibodies, have MN, probably due to a different target antigen discovered in the last ten years, such as EXT1/2, NELL1, Sema3B, NCAM1, PCDH7, HTRA1, and NTNG1 [86,87].

The remaining 20% recognize secondary causes, in particular infections, tumors, and other autoimmune diseases [87].

Experimental data from the Heymann nephritis rat model of human MN have shown that IgG antibodies in subepithelial immune deposits start complement activation and C5b-9-mediated damage of the podocyte. Although IgG can activate the classical pathway, there is also evidence that alternative pathway activation occurs in MN, which could occur because of absent, dysfunctional, or inhibited podocyte complement regulatory protein [88].

Increased plasma level of C3a and C3aR expression and deposition of C5b-9 in glomeruli have been found in MN patients [88].

The use of eculizumab versus placebo was attempted in a multicenter, double-blind study that found no difference in proteinuria after 16 weeks. More encouraging was the reduction in proteinuria, with open-label use of eculizumab for up to 1 yr in some patients [89].

Considering the role of complement, iptacopan versus rituximab has been evaluated. The study (NCT04154787) was terminated early because superiority of ipatcopan vs. rituximab in the reduction of p3roteinuria at 24 weeks was not possible to achieve. At the moment, despite the evidence of complement involvement of pathogenesis of MN, more studies are necessary to understand if anti-complement therapy may play a key role in the treatment of MN in the future. 

## 5. Conclusions

The role of drugs aimed at controlling complement activation in several parts of the complement cascade is already central in several kidney diseases. The pioneer drug was eculizumab, which made it possible to drastically change the prognosis of an otherwise fatal disease such as aHUS. To date, three drugs to treat kidney disease are already commercially available (eculizumab, ravulizumab, and avacopan), but several others are on the verge of commercialization (iptacopan, cemdisaran, danicopan, and pegtecatoplan). Moreover, a large number of studies aimed at improving the kidney prognosis of primary or secondary glomerulonephritis, kidney transplantation, and AKI are still ongoing. Nephrologists must learn to take a curious and enthusiastic look at these new therapeutic perspectives, which are revolutionizing nephrology.

## Figures and Tables

**Table 1:** Anti-complementary drugs and clinical trials in kidney disease. aHUS: atypical hemolytic uremic syndrome; C3G: C3 glomerulopathy; IgAN: IgA nephropathy; AAV: ANCA-associated vasculitis; MN: membranous nephropathy; LN: lupus nephritis; TA-TMA: transplanted associated thrombotic microangiopathy.

Drug	Disease	Clinical Trial	Primary Aims	Status
**ECULIZUMAB** Target: C5	aHUS	phase 2—NCT01194973	Percentage of patients with complete TMA response.	completed
	aHUS	phase 2—NCT00844545	1. Platelet count change from baseline to 26 weeks. 2. Percentage of patients with platelet count normalization. 3. Percentage of patients with hematologic normalization.	completed
**RAVULIZUMAB** Target: C5	aHUS	phase 3—NCT03131219	Percentage of complement inhibitor treatment-naïve participants with complete TMA response at week 26.	completed
	aHUS	phase 3—NCT02949128	Percentage of participants with complete TMA response at week 26.	completed
	LN–IgAN	phase 2—NCT04564339	Percentage change in proteinuria from baseline to week 26, assessed using 24-h urine collections.	recruiting
	IgAN	phase 3—NCT06291376	1. Change from baseline in proteinuria based on 24-h urine protein creatinine ratio at week 34. 2. Glomerular filtration rate over 106 weeks.	recruiting
**IPTACOPAN** Target: Factor B	aHUS	phase 3—NCT04889430	1. Percentage of participants with complete TMA response without the use of plasma exchange and anti-C5 antibody. 2. Long-term safety and efficacy evaluations.	recruiting
	aHUS	phase 3—NCT05795140	1. Number of participants with adverse events and serious adverse events. 2. Number of participants with abnormal safety laboratory parameters, vital signs, and electrocardiography.	recruiting
	aHUS	phase 3—NCT05935215	Percentage of participants free of TMA manifestation.	recruiting
	MN	Phase 2—NCT04154787	Ratio between baseline urine protein creatinine ratio and urine protein creatinine ratio at 24 weeks of treatment.	completed
	IgAN	phase 3—NCT04557462	Number and percentage of participants with adverse event and serious adverse event and with abnormalities in clinical laboratory evaluations.	recruiting
	LN	phase 2—NCT05268289	Proportion of patients achieving complete kidney response at week 24 in the absence of kidney flares.	recruiting
	C3G	phase 3—NCT04817618 (APPEAR-G3G)	1. Log-transformed ratio to baseline in urine protein creatinine ratio. 2. Change from baseline in log-transformed urine protein creatinine ratio at 12 months.	recruiting
	AAV	phase 2—NCT06388941	Sustained remission through week 48 defined as complete remission at week 24 without major relapse up to week 48.	recruiting
**ANX009** Target: C1q	LN	phase 1B—NCT05780515	Number of participants with treatment-emergent adverse events.	completed
**CROVALIMAB** Target: C5	aHUS	phase 3—NCT04958265	Percentage of participants with complete TMA response (naive cohort only).	recruiting
	aHUS	phase 3—NCT04861259	Percentage of participants with complete TMA response.	recruiting
**NOMACOPAN** Target: C5	TA-TMA	phase 3—NCT04784455	RBC transfusion independence or urine protein creatinine ratio ≤ 2 mg/mg maintained over ≥28 days immediately prior to any scheduled clinical visit up to week 24.	terminated
**PEGCETACOPLAN** Target: C3	TA-TMA	phase 2—NCT05148299	PK parameter concentration maximum, time of maximum measured serum concentration.	completed
	IgAN	phase 2—NCT03453619	Proteinuria reduction from baseline to week 48, based on urinary protein-to-creatinine ratio.	completed
	C3G	phase 3—NCT05067127 (VALIANT)	The log-transformed ratio of urine proteine creatinine ratio at week 26 compared to baseline.	active, not recruiting
	C3G	phase 3—NCT05809531 (VALE)	Proportion of participants with a reduction in urine protein creatinine ratio of at least 50% from the pretreatment value over time.	active, not recruiting
**CEMDISIRAN** Target: C5	IgAN	phase 2—NCT03841448	Percent change from baseline in urine protein creatinine ratio at week 32.	completed
**AVACOPAN** Target: C5R	AAV	phase 3—NCT02994927 (ADVOCATE)	Percentage of subjects achieving sustained disease remission at week 52.	completed
	IgAN	phase 2—NCT02384317	1. Change in slope of urinary proteine creatinine ratio from the 8-week renin angiotensin aldosteron blocker run-in period to the 12-week treatment period. 2. Number of participants with advers events.	completed
	C3G	phase 2 -NCT03301467 (ACCOLADE)	Change from baseline to week 26 in the C3G Histologic Index for disease activity—subjects with elevated C5b-9.	completed
**VILOBELIMAB** Target: C5a	AAV	phase 2—NCT03712345	Number and percentage of participants with at least one treatment-emergent adverse event per treatment group.	completed
**IONIS-FB-LRX** Target: Factor B	IgAN	phase 2A—NCT04014335	Percent reduction in 24-h urine protein excretion.	completed
**NM8074** Target: Factor B	IgAN	phase 2—NCT06454110	Change from baseline or percent change from baseline in urine protein-to-creatinine concentration ratio.	Not yet recruiting
	C3G	phase 1b—NCT05647811	1. Monitoring for incidence of adverse events/serious adverse events. 2. Change from baseline or percent change from baseline in urine protein to creatine concentration ratio.	not yet recruiting
**NARSOPLIMAB** Target: MASPs	IgAN	phase 2—NCT02682407 (ARTEMISAN-IgAN)	1. Proportion of subjects with treatment-related adverse events. 2. Change from baseline in serum and urine complement component levels.	completed
	IgAN	phase 3—NCT03608033	Change from baseline in 24-h urine protein excretion in IgAN assessed at 36 weeks from baseline.	completed
**ZALTENIBART** Target: MASPs	C3G	Phase 2—NCT06209736	To assess 5 mg/kg IV administration at 4-week intervals.	recruiting

## Data Availability

No new data were created or analyzed in this study. Data sharing is not applicable to this article.
